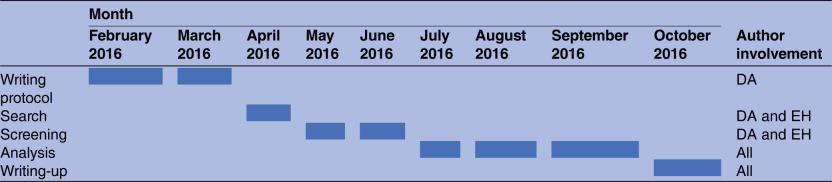# Mapping the progress and impacts of public health approaches to palliative care: a scoping review protocol

**DOI:** 10.1136/bmjopen-2016-012058

**Published:** 2016-07-12

**Authors:** Daryll Archibald, Rebecca Patterson, Erna Haraldsdottir, Mark Hazelwood, Shirley Fife, Scott A Murray

**Affiliations:** 1 Scottish Collaboration for Public Health Research & Policy (SCPHRP) Centre, University of Edinburgh, Edinburgh, UK; 2 Scottish Partnership for Palliative Care, Edinburgh, UK; 3 St Columba’s Hospice, Edinburgh, UK; 4 NHS Lothian, Edinburgh, UK; 5 Primary Palliative Care Research Group, Centre for Population Health Sciences, The Usher Institute of Population Health Sciences and Informatics, The University of Edinburgh

**Keywords:** PUBLIC HEALTH, PALLIATIVE CARE, HEALTH PROMOTION

## Abstract

**Introduction:**

Public health palliative care is a term that can be used to encompass a variety of approaches that involve working with communities to improve people's experience of death, dying and bereavement. Recently, public health palliative care approaches have gained recognition and momentum within UK health policy and palliative care services. There is general consensus that public health palliative care approaches can complement and go beyond the scope of formal service models of palliative care. However, there is no clarity about how these approaches can be undertaken in practice or how evidence can be gathered relating to their effectiveness. Here we outline a scoping review protocol that will systematically map and categorise the variety of activities and programmes that could be classified under the umbrella term ‘public health palliative care’ and highlight the impact of these activities where measured.

**Methods and analysis:**

This review will be guided by Arksey and O'Malley's scoping review methodology and incorporate insights from more recent innovations in scoping review methodology. Sensitive searches of 9 electronic databases from 1999 to 2016 will be supplemented by grey literature searches. Eligible studies will be screened independently by two reviewers using a data charting tool developed for this scoping review.

**Ethics and dissemination:**

This scoping review will undertake a secondary analysis of data already collected and does not require ethical approval. The results will facilitate better understanding of the practical application of public health approaches to palliative care, the impacts these activities can have and how to build the evidence base for this work in future. The results will be disseminated through traditional academic routes such as conferences and journals and also policy and third sector seminars.

**Strengths and limitations of this study**

- No previous work has been carried out to systematically map and categorise the wide variety of activities and programmes that could be classified under the umbrella term ‘public health palliative care’.
- This work will shed much needed light on the potentially wide-ranging impact of public health palliative care activities and explore ways in which impact has been or might be measured.
- A scoping review can help to understand whether the palliative care community instigate this type of engagement activity or whether other organisations (eg, local charities, community groups and arts organisations) are involved in activities that constitute public health palliative care but which they have not labelled as such.
- No formal quality assessment of included studies is performed. This is because scoping reviews provide a map of what evidence has been produced as opposed to seeking only the best available evidence to answer a particular policy and practice-related question.

## Introduction

A social model of health recognises that influences on people's health are far broader than disease and injury and that they include social, cultural, environmental and economic factors, looking further than lifestyles and behaviour, and recognising that social change can be a prerequisite for health.^1^ In recent decades, there has been growing recognition that a social model of health is helpful in understanding how to improve people's experiences of death, dying and bereavement. Kellehear^2^ has been a key influence in academic and practical work in this area, developing the Health Promoting Palliative Care (HPPC) model in the late 1990s. As its name suggests, HPPC brings together two perspectives—health promotion and palliative care—to focus on improving experiences of death, dying and bereavement.

Kellehear's work illustrates how the principals of the World Health Promotion Guidelines contained in the Ottawa Charter^3^ can be applied to palliative and end-of-life care, widening the traditional service-oriented palliative care model by building policies, creating supportive environments, strengthening community actions, developing personal skills and reorienting health services to support the experiences of dying, death and loss. The linkage of palliative care and health promotion is a good example of the ‘new public health’ that became prominent in the 1980s which emphasised the importance of the social determinants of health and the active role of individuals in securing their own health.^4^

As the field has developed over recent years, several terms have emerged to describe approaches that are related to or similar to HPPC. In 2014, Sallnow and Paul^5^ attempted to develop some conceptual clarity around use of these terms:

A range of terms have now entered the discourse, including ‘public health approaches to palliative care’ (Conway, 2008), ‘compassionate cities’ (Kellehear, 2005), ‘compassionate communities’ (Abel, Bowra, Walter, & Howarth, 2011) and ‘health promoting palliative care’ (Kellehear, 1999). Such initiatives serve to: improve the relevance of the services offered; develop skills, knowledge and capacity in communities; support coping and resilience in the face of death, dying and loss. (p. 232)

In this relatively new and rapidly developing field, there remains a lack of clarity regarding definitions and use of terminology, and it is outside the remit of this review to address this issue in detail. With the establishment of two new organisations, Public Health Palliative Care International and Public Health Palliative Care UK in 2015, ‘public health palliative care’ is a relatively recently devised term which is growing in popularity.^6^ Recognising that no single term is perfect, within this article, we use the term ‘public health palliative care’ as an umbrella term to encompass the range of approaches and initiatives relevant to this review, drawing on the conceptualisation by Sallnow and Paul referenced above.

In recent years, approaches to public health palliative care have attracted interest and gradually gained momentum within UK health practice and policy, and among those who are part of and responsible for palliative care services.^7^ There is general consensus that public health palliative care approaches can complement and go beyond the scope of formal service models of palliative care. However, there is not widespread clarity about how these approaches can be undertaken in practice or how evidence can be gathered relating to the effectiveness of these approaches.

No previous work has been carried out to systematically map and categorise the wide variety of activities and programmes that could be classified under the umbrella term ‘public health palliative care’, to understand the potentially wide-ranging impact of these activities or to explore ways in which impact has been or might be measured.

Despite the rapid international growth of interest in approaches to public health palliative care, few overviews of literature on these issues are available to date. Rosenberg and Yates^8^ conducted a critical review of literature relevant to the conceptual foundations of HPPC that explored the early considerations regarding the convergence of palliative care and health promotion. Sallnow *et al*^4^ undertook a systematic review of the evidence relating to the impact of a new public health approach to end-of-life care, specifically as this applies to efforts to strengthen community action.

This article outlines the protocol for a scoping review which aims to:

1. Systematically map and categorise the wide variety of activities and programmes that could be classified under the umbrella term ‘public health palliative care’.
2. Document the impact of these activities where impact has been measured.

A review of this kind has the potential to shed light on whether the palliative care community are the main instigators of this type of engagement activity, or whether other organisations (eg, local charities, community groups and arts organisations) are involved in activities that constitute public health palliative care but which they have not labelled as such. It will set out some of the different understandings, interpretations and approaches which could fall under the umbrella of public health palliative care. It can also provide valuable information about the range of ways that the theory of public health palliative care has been applied in practice in various settings to inform how the concept is understood in practice as well as informing future work in this area. There is also the potential for future work which more fully explores how the impact of activities can be evaluated and what barriers and enablers exist regarding building the evidence base for this field of work.

## Methods

Various approaches are available for reviewing and synthesising literature; however, given the aims listed above, a scoping review is the most suitable review method to deploy in this case. This can be said as the intention of this review is to produce an overall map of what evidence has been produced as opposed to the approach associated with systematic reviewing where the best evidence available is sought to answer a tightly defined question related to policy and/or practice.^9^

Scoping reviews are thus broader in nature than systematic reviews in that they provide an overview of existing evidence regardless of quality. Scoping reviews, therefore, allow researchers to examine the extent, range and nature of research activity in their chosen area. Despite no formal quality assessment being undertaken, scoping reviews nevertheless apply a comprehensive and systematic approach to mapping the literature, key concepts, theories, evidence and research gaps in a field using broadly framed questions.^9^

This scoping review will conform to the five-stage framework laid out by Arksey and O’Malley;^10^ however, in setting out the plan of the review in this way, we will also draw on more recent refinements to Arksey and O’Malley's framework by Levac *et al*^11^ and the Joanna Briggs Institute.^9^

### Stage 1: Identifying the research questions

The first stage in the process of conducting a scoping review is to identify the research question(s) for the study and to link the question with purpose of the study.^10^ ^11^ With that in mind, we developed a series of research questions related to the aims of the study. However, as the process of conducting a scoping review is often iterative, requiring a reflexive approach to each stage as the researcher becomes increasingly familiar with the literature, there is a possibility that revisions may be made to the research questions. Six research questions were identified to guide the scoping review. These questions were developed via a series of research team meetings:

1. What programmes have been carried out and are presented in the social science and medical literature around public health palliative care?
2. What barriers and facilitators to implementing programmes are identified in the social science and medical literature around public health palliative care?
3. What programmes have been evaluated in the social science and medical literature around public health palliative care and how have they been evaluated?
4. What impacts are reported in the social science and medical literature around public health palliative care?
5. What are the key gaps in the social science and medical literature around public health palliative care?
6. What are the target populations being addressed in the social science and medical literature around public health palliative care?

### Stage 2: Identifying relevant studies

Sallnow and Paul^5^ provide a conceptualisation which acts as a useful basis for identifying relevant work for this scoping review:

Such initiatives serve to: improve the relevance of the services offered; develop skills, knowledge and capacity in communities; support coping and resilience in the face of death, dying and loss. (p. 232)

In consultation with a senior medical librarian at the University of Edinburgh, we developed a working framework for a search strategy (see online supplementary appendix 1). The design of the search strategy was underpinned by key inclusion criteria (see box 1). These criteria were categorised according to the broad Population—Concept—Context (PCC) mnemonic recommended by the Joanna Briggs Institute for scoping reviews^9^ as a less restrictive alternative to the PICO (Population, Intervention, Comparator and Outcome) mnemonic recommended for systematic reviews.

Box 1 **Inclusion criteria**

Population

- Human participants
- Any age
- Any sex

Concept

- Any public health palliative care initiative carried out between 1999 and 2016

Context

- Research articles are limited to developed countries (and regions) including UK, Canada, USA, Continental Europe, Australia and New Zealand where contemporary societal attitudes to death and dying may be comparable
- All settings considered
- Original research articles (any methods) and review articles including: systematic reviews, meta-analyses, meta-syntheses, narrative reviews, mixed-methods reviews, qualitative reviews and rapid reviews

10.1136/bmjopen-2016-012058.supp1 Supplementary appendix

#### Search strategy

The search strategy will follow the three-step process recommended by the Joanna Briggs Institute.^9^ The first of these steps has been undertaken and involved a limited preliminary search of one online database relevant to the topic (Ovid MEDLINE). This search resulted in 6787 studies.

The second step will contain an analysis of the text words contained in the title and abstract of retrieved papers, and of index terms used to describe the articles. A second search using all identified keywords and index terms will then be undertaken across all included databases. These databases will include Ovid EMBASE, EBSCO CINAHL, EBSCO PsycINFO, Proquest, Applied Social Sciences Index and Abstracts, Proquest Education, Resources Information Center, OCLC Anthropology Plus, Ovid British Nursing Index, Social Sciences Citation Index and Conference Proceedings Citation Index—Social Science & Humanities.

The third and final step will check the reference lists of all identified reports and articles for additional studies. Grey literature searches will also be undertaken to identify any non-indexed literature of relevance to this review. The final included studies will be held stored using a reference management software package and duplicates will be removed.

### Stage 3: Study selection

The study selection process will be implemented over two stages. The first stage will involve the review of titles by one reviewer (DA) to determine study eligibility based on the above stated inclusion and exclusion criteria. For example, foreign language titles or titles that indicate a study was carried out in an ineligible country will be removed. Titles will be screened as ‘included’, ‘excluded’ or ‘uncertain’. Should uncertainty arise with a title in the first stage, the citation will be considered in the second stage.

The second stage of the selection process will see two reviewers (DA and EH) apply the inclusion criteria to all abstracts. Should differences arise, the reviewers will consult with a third reviewer to reach consensus. When consensus is not reached, those articles will be included in the review.

To recap, a formal assessment of the quality of included studies will not be undertaken as scoping reviews aim to provide a map of what evidence has been produced rather than seeking only the best available evidence to answer a particular question related to policy and practice.

### Stage 4: Charting the data

The process of data extraction in scoping reviews is termed ‘charting’ the results.^8^ The charting process aims to generate a descriptive summary of the results that corresponds to the aims and research questions of the scoping review. A draft charting form (see box 2) has been developed at the protocol stage to aid the collection and sorting of key pieces of information from the selected articles.

Box 2 **Draft data charting form**

1. *Bibliographic information*

Study ID

Article title

Extracted by

Checked by

Type of publication (journal article, book chapter, grey literature)

Country

2. *Researcher details*

Authors and affiliations (list as presented on paper)

3. *Aims and methods*

Study aims/objectives

Methodology

Methods

4. *Scoping review PCC*

Population

Concept (interventions/programmes and outcomes assessed)

Context

5. *A priori themes (does the paper report data relating to the following?)*

- Social difficulties around death, dying, loss or care
- Reducing harms associated with death, dying, loss or care (eg, isolation/loneliness)
- Early interventions along the journey of death, dying, loss or care?
- Changes to settings/environments
- Participatory approaches
- Sustainable approaches
- Evaluability

6. *Emergent themes (does the paper report on any further issues not related to the above that might be of interest to this review?)*

a. 

b. 

c. 

d. 

Data to be extracted from the included studies will include standard information (such as author, year of publication and study objectives). In addition, further information pertaining to the key features of programmes and activities that promote ‘public health palliative care’ will be searched for in the included studies. This additional information takes the form of a priori categories that incorporate Kellehear's ‘Big 7’ checklist,^12^ which is a seven-point checklist to assess how well a programme/initiative matches the criteria of HPPC. However, additional categories may emerge during the data collection, and the data extraction form will include a category for reviewers to record emergent themes that will be discussed and refined during research team meetings. This may be further refined at the review stage and the charting form updated accordingly.

### Stage 5: Collating, summarising and reporting the results

The central challenges to undertaking a scoping review centre on determining a framework for presenting a narrative account.^13^ With that said, the strategy of reporting results from this review will draw on recent innovations in reporting scoping review results, such as from Halas *et al*^13^ and Nelson *et al*.^14^ Both of the aforementioned studies advocate using a modified version of the Preferred Reporting Items for Systematic Reviews and Meta-Analysis (PRISMA)^15^ to present results from the search process. We will also modify the PRISMA checklist, specifically by incorporating the elements of the checklist that are congruent with the underpinnings of scoping review methodology while removing points that are not, such as those points that relate to bias. Drawing further on the work of Levac *et al*^11^ and Nelson *et al*,^14^ we will also present a numerical overview of the amount, type and distribution of the included studies. The central section of the review will comprise a thematic summary of the findings that relates the a priori and emergent categories extracted from the included studies to the research questions stated above.

## Conclusion

Scoping reviews can be complex to undertake; however, an a priori protocol will help in the process of preparing for such a review in order to provide an approach that offers clarity, strength and transparency to avoid problems occurring during the undertaking of the review. The review will have relevance to a variety of audiences including researchers, clinicians and policymakers interested in better understanding the practical application of public health palliative care, the impacts these activities can have and how to build the evidence base for this work in future. The study research team includes experts in public health palliative care from academia, NHS Scotland and the third sector. Table 1 shows the timeline for study completion.

**Table 1:** Timeline for protocol and scoping review