# Supplementary material for: Mapping the progress and impacts of public health approaches to palliative care: a scoping review protocol
Source: BMJ Open. 2016 Jul 12;6(7):e012058. doi: 10.1136/bmjopen-2016-012058 (PMC4947716; doi:10.1136/bmjopen-2016-012058)
Supplement: Supplementary appendix [file bmjopen-2016-012058supp_appendix.pdf]

APPENDIX 1: Search Strategy for Ovid Medline

1. Health promoting palliative care.mp.
2. public health approaches to palliative care.mp.
3. (public health approach* adj3 (palliative or end of life)).mp. [mp=title, abstract, original title, name of substance word, subject heading word, keyword heading word, protocol supplementary concept word, rare disease supplementary concept word, unique identifier]
4. Public health palliative care.mp.
5. end of life care.mp.
6. compassionate cities.mp.
7. compassionate communities.mp.
8. compassionate commun*.mp. [mp=title, abstract, original title, name of substance word, subject heading word, keyword heading word, protocol supplementary concept word, rare disease supplementary concept word, unique identifier]
9. palliative care/ or terminal care/ or hospice care/
10. health promotion/ and (end of life or hospice or terminal or palliative).mp. [mp=title, abstract, original title, name of substance word, subject heading word, keyword heading word, protocol supplementary concept word, rare disease supplementary concept word, unique identifier]
11. or/1-9
12. Attitude to Death/
13. bereavement/ or grief/ or loneliness/
14. community development.mp. or Social Change/
15. community engagement.mp.
16. social support.mp. or Social Support/
17. (program* or intervention* or campaign* or strateg*).mp. [mp=title, abstract, original title, name of substance word, subject heading word, keyword heading word, protocol supplementary concept word, rare disease supplementary concept word, unique identifier]
18. death education.mp.
19. or/11-17
20. 10 and 18
21. limit 19 to english language
22. limit 20 to yr="1999 -Current"
23. limit 21 to journal article
24. 11 or 12 or 13 or 14 or 15 or 17
25. 10 and 23
26. limit 24 to journal article
